# Case of Pseudo-Membranous Laryngitis

**Published:** 1849

**Authors:** C. D. Meigs


ARTICLE I.

*Case of Pseudo-Membranous Laryngitis.* By Dr. C. D. Meigs.

A child, æt. four, becoming affected with difficult and noisy respiration, was placed under the care of a homœpathic practitioner; the parents having lost already a child from the croup, recognized in this the same symptoms as were observed in the former case, and suggested to the medical attendant that the child was laboring under that disease, but this he declared was not the case, but rather thought that the attack would turn out to be one of measles. The child, however, grew worse and worse; no eruption appeared upon the skin, and at the end of two weeks, the respiration having become increased in difficulty and attended with a distinct croupy sound, while the voice of the child was nearly extinct, the parents of the child became alarmed and sent for Dr. Meigs. His son, Dr. J. F. Meigs, immediately saw the patient and found it in advanced stage of genuine membranous croup, attended with symtoms of the most violent character; an extensive depositon of membranous matter appeared to have taken place, and the case was looked upon as almost hopeless. With the view, however, of affording, if possible, some relief to the extreme difficulty of breathing, the doctor directed the application of five or six leeches to the throat on each side of the trachea. Dr. C. D. Meigs now saw the child, and considered it to be in the most imminent danger. The croupal symptoms were intense. Upon auscultation, not the slightest respiratory mumur could be detected in any part of the chest, giving the idea of an individual laboring under complete hepatization of both lungs. The air passed into the lungs with the greatest difficulty, the respiratory effort being prolonged to an extent beyond what the doctor recollects to have ever before witnessed. The child was extremely restless, its head was thrown back upon the spine, and every moment strangulation seemed imminent. A half-ounce of powdered alum was directed, and one drachm given to the child at intervals of twenty minutes, until emesis was produced, which did not occur until after the fourth dose. This was rather an uncommon occurrence, vomiting being generally produced by a single dose of alum; it evidently indicated a torpid state of the nervous mass, the result of the great change produced in the blood, in consequence of the imperfect performance of the respiratory function. No nausea or prostration followed the action of the emetic.

Early the next morning, found the child laboring under the most distressing difficulty of respiration. The surface, and particularly the face, lips, and tongue, were of a blue color, and nearly all the symptoms of asphyxia were present. Dr. Meigs considered that death was inevitable, but still the opeation of tracheotomy, though a forlorn hope, presented itself as the only possible means of relief. This was stated to the parents, who consented that it should be tried. Accordingly at 11 o'clock the operation was performed by Dr. Pancoast. After laying bare the trachea, he divided the second, third and fourth cartilaginous rings; immediatly upon opening the trachea, a discharge took place of mucus, mixed with blood and portions of plastic lymph. In forty seconds, the child breathed with great freedom, Instead of inserting a tube in the usual manner through the opening into the trachea, Dr. Pancoast secured the open state of this by cutting from the trachea an elliptical portion of cartilage, thus leaving an oval opening into the tube, somewhat larger than that of the two nostrils; while the edges of the incision through the soft parts were kept asunder by a leaden wire, which, passing around the neck, had the hooked ends of its two free extremities inserted on each side of the wound. The next day the child was up and running about. In a few days the edges of the incision in the neck were brought together, the wound rapidly healed, and the child within a suprisingly short period, recovered perfectly without a single disagreeable symptom occurring.—*Trasanct. Philad. College of Physicians*, 1848.

